# Suicide risk among female breast cancer survivors: A population–based study

**DOI:** 10.3389/fonc.2022.986822

**Published:** 2022-11-24

**Authors:** Jian Shi, Yongping Yang, Yu Guo, Wu Ren

**Affiliations:** ^1^ Department of General Surgery, The Second Hospital of Jilin University, Changchun, Jilin, China; ^2^ Department of Obstetrics and Gynecology, Tongji Hospital, Tongji Medical College, Huazhong University of Science and Technology, Wuhan, Hubei, China

**Keywords:** Breast cancer, suicide, molecular subtype, risk factors, nomogram

## Abstract

**Background:**

Breast cancer is the most common cancer type for females and has the highest relative number of suicide cases among female-specific cancers. This study aimed to demonstrate suicide rates and changing trends and to identify risk factors for suicide among female breast cancer survivors.

**Methods:**

Data were derived from the surveillance, epidemiology, and end results database for women diagnosed with breast cancer from 2000 to 2017. Mortality rate and standardized mortality ratios (SMRs) were calculated to describe the incidence rate and trend of suicide among female breast cancer survivors. Gray’s test and cumulative incidence function (CIF) curves were used to assess difference of cumulative suicide incidence in subgroups. Multivariate Fine-Gray competing risk model was used to identify risk factors for individual survivors and nomogram model was used to estimate the probability of suicide.

**Result:**

There were 414 suicide cases among 638,547 female breast cancer survivors observed for 5,079,194 person-years, and the suicide rate and SMRs gradually increased with the year of diagnosis. Female breast cancer survivors had a higher risk of suicide than the general population (SMR = 1.19; 95% CI (1.08–1.31)). Based on the result of Fine-Gray competing risk models, age group (50-70 vs <50: HR=0.65, 95% CI:0.52-0.80; >70 vs <50: HR=0.22, 95% CI:0.15-0.32), race/ethnicity (black vs white: HR= 0.20, 95% CI: 0.11-0.36; other race vs white: HR= 0.67, 95% CI: 0.46-0.97), marital status (separated vs married: HR= 1.50, 95% CI: 1.16-1.94; single vs married: HR= 1.70, 95% CI: 1.31-2.20), stage (distant vs regional: HR= 0.30, 95% CI: 0.14-0.63), radiotherapy (Yes vs No/Unknown: HR= 0.62, 95% CI: 0.49-0.77), and molecular subtypes (HER-2 vs Luminal B (HR= 2.53, 95% CI: 1.10-5.82), TNBC vs Luminal B (HR= 2.11, 95% CI: 1.01-4.42)) were independent predictors of suicide among female breast cancer patients. A nomogram was constructed to predict the suicide probability for individual survivors with a C-index of 0.62 (95%CI: 0.59-0.66).

**Conclusion:**

Female breast cancer survivors with younger age (less than 50 years old), white race, unmarried status, regional stage, HER-2 or TNBC subtype, and no radiotherapy performed were more likely to commit suicide. The clinicians and family members should pay more attention to patients with high risk factors of suicide to decrease the mortality rate.

## Introduction

Suicide, a death caused by intentional self-directed injury, is a complex global public health issue (1, 2). It is the 18th leading cause of death in the world and the 10th leading cause of death in the United States (US) (3–5). There were 817 000 suicide cases in 2016 reported by the Global Burden of Disease group, which accounted for 1.49% of the total deaths (6). While suicide rates have declined over recent decades in most countries, the rate in the US has increased by 1.5% annually since 2000 (3, 5).

Cancer is one of the leading cause of death, and there was an estimated 19.3 million new cancer cases and almost 10.0 million cancer deaths occurred in 2020 (7). Traditionally, the primary goal of cancer treatment was to prolong survival, including measures for improved screening, early diagnosis and treatment. As cancer is considered to be associated with long-term trajectory of both treatment and recovery, survivors would experience physical, financial and emotional burden (8). Despite recent improvements in rates of cancer survival, mortality from suicide among people with cancer continues to be higher than the general US population (2, 9–11). It is surprising that suicide rates among cancer survivors are generally thought to be twice as high as that of the general US population (12). A current study revealed that the cancer survivors had a suicide standardized mortality ratio (SMR) of 4.44 compared to the general population (10). Social (age, sex, economic status, and marital status) and clinical (depression, cancer site, and subtypes) risk factors are all associated with suicide among cancer survivors (13). As it’s difficult to detect suicide attempts, identifying and targeting subgroups of cancer survivors at high risk of suicide is very important. What’s more, given the difference existing in site-specific cancers, we need to focus on specific cancer survivors to estimate suicide burden and identify risk factors.

Female breast cancer is the most common cancer type in the world, accounting for the highest number of new cases and the fourth highest death among all cancer types in 2020 (7). Despite all of the attention being paid to breast cancer survivorship, recent years have shown a very slight improvement in quality of life (14). Previous study reported that age, race, marital status, and undergoing surgery were independent risk factors for suicide of breast cancer survivors (15). However, male and female sexes were combined in this study and breast cancer molecular subtypes were not included in the study. And the regression method for identifying risk factors used in that study could not take survival time into consideration. Therefore, the purpose of this study was to compare suicide rates among female breast cancer survivors with that in the general population as well as in survivors of other cancers, to characterize changing trends of suicide among female breast cancer survivors, and to discover risk factors of suicide relevant to survival time using data from the Surveillance, Epidemiology, and End Results (SEER) database.

## Methods

### Data collection

The SEER database, which includes data on roughly 28% of the US population, is the largest publicly accessible cancer database at the moment (16). Data on a patient’s demographics and clinical features, such as sex, age at diagnosis, year of diagnosis, race, marital status, tumor grade and stage, histological type, treatment, and survival time, are available in the SEER database. Data used in this study were derived from the SEER Program 17 registries using the SEER Stat software (version 8.4.0). The SEER Program 17 registries provided data on survivors diagnosed from 2000 to 2019. Population and mortality data of the general US population were gathered from the National Center for Health Statistics in 1969–2019 and acquired *via* the SEER Stat software. We used data on survivors diagnosed from 2000 to 2017 since different standards of clinical features, such as pathological staging and histological grading standards, were in use at different times.

### Inclusion and exclusion criteria

Data were included following these criteria: 1) female patients, 2) primary site: breast (C50.0 – C50.9), 3) malignant behavior, 4) only one primary cancer, 5) histology type: 8500/3-8549/3. Patients were excluded if the diagnosis was made at autopsy or from the death certificate, and those without definite data on age at diagnosis, survival time and race information were also excluded in this study. Available data about demographic characteristics from the SEER database included age at diagnosis, race/ethnicity, year of diagnosis, marital status, and residential areas. Clinical characteristics included histologic and molecular subtype, stage, grade, treatment (surgery, chemotherapy, and radiotherapy), follow-up time, and cause of death. If the cause of death feature was recorded as “ Suicide and Self-Inflicted Injury (50220)”, the patient was assumed to have committed suicide. Age was categorized into three groups: 0-49 years old, 50-69 years old, and 70+ years old. Racial/ethnicity was categorized as white, black, and other races/ethnicity (American Indian/Alaskan Native, Asian/Pacific islanders). Year of diagnosis was categorized into four periods: 2000-2004, 2005-2009, 2010-2014, and 2015-2017. Marital status included married (married and having domestic partner), single (never married), and separated (separated, divorced, and widowed). Breast cancer was categorized into four histological subgroups according to the International Classification of Disease for Oncology third revision (ICD-O-3) codes: infiltrating duct cancer, infiltrating lobular cancer, infiltrating duct and lobular cancer, and other type. Surgery types were categorized into four groups according to SEER program surgery coding and staging manual: None surgery, breast-conserving surgery (BCS), mastectomy, and Unknown. Data after 2010 included breast cancer molecular subtypes which were clarified according to the expression of hormone receptor (HR) and HER-2 gene. Molecular subtypes included Lumina A (HR+, HER-2 (–)), Lumina B (HR(+), HER-2(+)), HER-2 (HR (–), HER-2(+)), and triple negative breast cancer (TNBC) (HR (–), HER-2 (–)).

### Statistical analysis

The majority of analyses were completed using data on survivors diagnosed from 2000 to 2017. Only the analyses that included molecular subtypes were performed using data on survivors diagnosed from 2010 to 2017. Suicide rate among female breast cancer patients was counted according to reported suicides per 100,000 person-years of follow-up. The chi-square test was used to compare suicide rates among patients in different groups. SMRs were calculated by comparing these suicide rates to the US population suicide rates at the National Center for Health Statistics, and adjusted according to age, race, and sex in the US population during the same period. SMRs were measured as the ratio of reported suicides in our cohort to expected suicide counts of overall population. The 95% confidence interval (CI) of the SMR was calculated using Byar’s approximation. Gray’s test and cumulative incidence function (CIF) curves were used to describe difference of suicide probability in subgroups. Multivariable Fine-Gray competing risk models had been conducted to determine crude and adjusted hazard ratios (HRs) as well as 95% CI, to identify underlying suicide-related risk factors. The resulting multivariate Cox regression model was used to calculate risk scores and build the final nomogram prediction model. The Harrell C-index was used to indicate the discrimination and the calibration plot curve was adopted to assess the calibration of the nomogram model. Survival time recorded as 0 month in the SEER database was converted to one-half of a month according to accepted epidemiologic practices. In this study, data were analyzed using SEER Stat and the R program (Version 3.6.3), and a p-value of < 0.05 was considered to be statistically significant.

## Result

### Incidence of suicide among female breast cancer survivors

**Figure 1:**
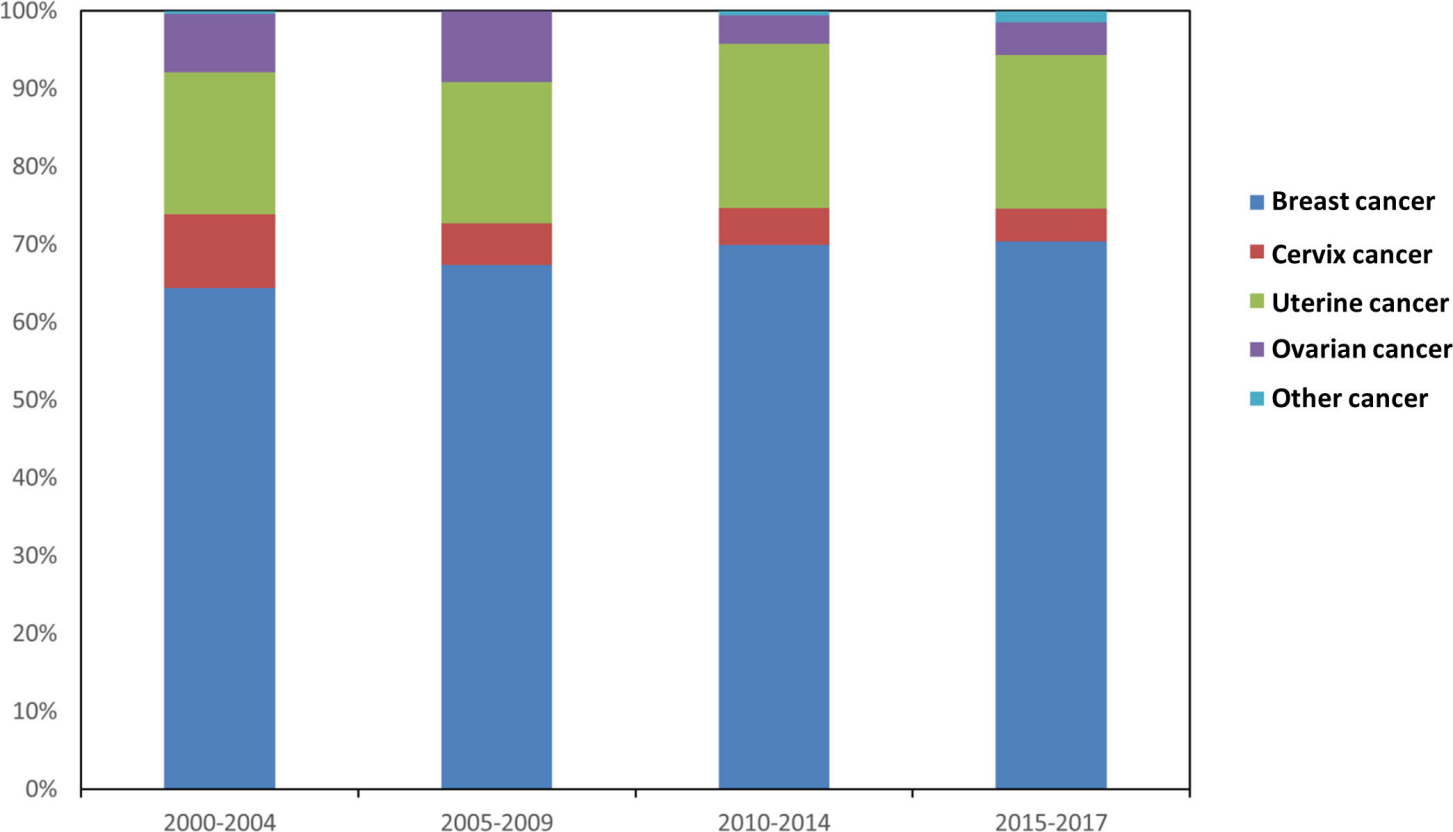
Relative frequency of suicide cases among survivors with different types of female-specific cancer diagnosed from 2000 to 2017.

A total of 414 suicide cases occurred among 638,547 female breast cancer survivors, followed by 5,079,193.52 person-years. The suicide rate and SMRs gradually increased from survivors diagnosed in 2000-2004 (mortality rate: 6.88 per 100,000 person-years; SMR= 1.11, 95% CI (1.08–1.31)) to survivors diagnosed in 2015-2017 (mortality rate: 10.99 per 100,000 person-years; SMR= 1.45; 95% CI (1.06-1.95)). The suicide rate was 8.15/100,000 person-years and women with breast cancer had a 19% higher risk of suicidal death (SMR=1.19; 95% CI (1.08–1.31)) compared to the general US population with the same distribution of age, sex, and race. During these four time periods, the relative frequency of suicide cases for breast cancer survivors ranked first among all female-specific cancer survivors and held an increasing trend (**Figure 1**).

### Suicide incidence according to demographic characteristics

There were 162, 205, and 47 suicide cases in less than 50 years, 50 to 70 years, and over 70 years age groups, separately. The suicide rate and SMR in younger age group of less than 50 years are higher than that in the other two groups (**Table 1**). Compared to the general population, significantly higher incidence rate of suicide was observed in all these three groups. Mortality rate reached the peak at 30-40 years old (mortality rate: 11.57 per 100,000 person-years; SMR=1.54, 95% CI (0.97-1.96)) and gradually decreased with increasing age after 40 years old (**Table S1**). As for race difference, patients from white race had the highest risk of suicide (mortality rate: 8.96 per 100,000 person-years; SMR=1.17; 95% CI (1.06-1.30)). Higher suicide rates were observed in patients living in metropolitan areas (mortality rate: 8.25 per 100,000 person-years; SMR=1.21, 95% CI (1.09-1.34)) and in patients unmarried including single status (mortality rate: 12.43 per 100,000 person-years; SMR=1.80, 95% CI (1.43-2.23)) and separated status (mortality rate: 8.68 per 100,000 person-years; SMR=1.52, 95% CI (1.24-1.84)), separately.

**Table 1:** Suicide rates, according to demographic and clinical characteristics, female breast cancer survivors, 2000 to 2017.

Variable		No. of patients,n (%)	Person-years	No. of deaths,n (%)	Mortality rate (per 100,000 person-years)	*P*-value	SMR (95%CI)
Age group						<0.001	
	0-49	158,851 (24.88)	1,407,290.30	162 (39.13)	11.51		1.27 (1.08-1.48)
	50-69	315,463 (49.40)	2,615,481.89	205 (49.52)	7.84		1.16 (1.12-1.33)
	70+	164,233 (25.72)	1,056,421.32	47 (11.35)	4.45		1.10 (0.81-1.46)
Race/ethnicity						<0.001	
	White	514,479 (80.57)	4,163,009.26	373 (90.10)	8.96		1.17 (1.06-1.30)
	Black	65,743 (10.30)	461,529.23	12 (2.90)	2.60		1.33 (0.69-2.33)
	Other	58,325 (9.13)	454,655.02	29 (7.00)	6.38		1.43 (0.96-2.05)
Year of diagnosis						<0.001	
	2000-2004	151,075 (23.66)	1,842,166.10	128 (30.92)	6.88		1.11 (0.93-1.32)
	2005-2009	165,755 (25.96)	1,612,842.83	133 (32.13)	8.89		1.19 (1.00-1.41)
	2010-2014	191,818 (30.04)	1,214,596.28	108 (26.09)	9.47		1.20 (0.99-1.45)
	2015-2017	129,899 (20.34)	409,588.30	45 (10.87)	10.99		1.45 (1.06-1.95)
Residence						<0.001	
	Metropolitan	571069 (89.43)	4,554,867.15	376 (90.82)	8.25		1.21 (1.09-1.34)
	Non-metropolitan	67328 (10.54)	523,335.76	38 (9.18)	7.26		1.06 (0.75-1.45)
	Unknown	150 (0.02)	990.60	0	0.00		
Marital status						<0.001	
	Married	357,018 (55.91)	3,039,985.22	209 (50.48)	6.88		0.95 (0.82-1.08)
	Single	89,924 (14.08)	667,848.20	83 (20.05)	12.43		1.80 (1.43-2.23)
	separated	162,769 (25.49)	1,175,624.92	102 (24.64)	8.68		1.52 (1.24-1.84)
	Unknown	28,836 (4.52)	195,735.17	20 (4.83)	10.22		1.52 (0.93-2.35)
Laterality						<0.001	
	Left-primary	324,000 (50.74)	2,572,752.69	207 (50.00)	8.05		1.19 (1.03-1.35)
	Right-primary	313,386 (49.08)	2,501,945.41	206 (49.76)	8.23		1.20 (1.04-1.38)
	Unknown	1,161 (0.18)	4,495.42	1 (0.24)	22.24		3.46 (0.09-19.29)
Grade*						<0.001	
	I	120,883 (18.93)	1,012,544.77	73 (17.63)	7.21		1.07 (0.84-1.35)
	II	261,727 (40.99)	2,100,877.91	160 (38.65)	7.62		1.12 (0.95-1.30)
	III	212,665 (33.30)	1,611,708.73	153 (36.96)	9.49		1.36 (1.15-1.59)
	IV	5,475 (0.86)	53,567.72	5 (1.21)	9.33		1.39 (0.45-3.25)
	Unknown	37,797 (5.92)	300,494.39	23 (5.56)	7.65		1.18 (0.75-1.77)
Stage						<0.001	
	Localized	391,245 (61.27)	3,319,253.02	234 (56.52)	7.05		1.05 (0.92-1.19)
	Regional	205,357 (32.16)	1,622,489.87	163 (39.37)	10.05		1.41 (1.21-1.65)
	Distant	32,132 (5.03)	98,683.55	9 (2.17)	9.12		1.33 (0.61-2.52)
	Unknown	6,813 (1.07)	38,767.08	8 (1.93)	20.64		3.21 (1.39-6.32)
Surgery*						<0.001	
	None	45,540 (7.13)	163,204.28	29 (7.00)	17.77		2.77 (1.86-3.98)
	BCS	338,671 (53.04)	2,887,177.71	191 (46.14)	6.62		0.98 (0.85-1.13)
	Mastectomy	252,188 (39.49)	2,015,933.27	190 (45.89)	9.42		1.34 (1.16-1.55)
	Unknown	2,148 (0.33)	12,878.26	4 (0.97)	31.06		4.67 (1.27-11.95)
Chemotherapy						0.346	
	No/Unknown	364,060 (57.01)	2,831,433.85	218 (52.66)	7.70		1.23 (1.07-1.40)
	Yes	274,487 (42.99)	2,247,759.67	196 (47.64)	8.72		1.15 (1.00-1.33)
Radiotherapy						<0.001	
	No/Unknown	303,600 (47.55)	2,354,005.79	248 (59.90)	10.54		1.57 (1.38-1.78)
	Yes	324,947 (52.45)	2,725,187.73	166 (40.10)	6.09		0.88 (0.75-1.02)
ALL		638,547	5,079,193.52	414	8.15		1.19 (1.08-1.31)

*SMR, standardized mortality ratio; CI, confidence intervals; Grade I, Well differentiated; Grade II, Moderately differentiated; Grade III, Poorly differentiated; Grade IV, Undifferentiated; BCS, breast-conserving surgery. P-value was calculated using the chi-square test.

### Suicide incidence according to clinical characteristics

Survivors with left sided breast cancer had a similar suicide rate and SMR compared to right sided breast cancer survivors. Survivors with high-grade breast cancer (III and IV) had higher suicide rates than survivors with low-grade (I and II) breast cancer. Survivors with regional and distant breast cancer had higher suicide rates (SMR, 1.41; 95% CI(1.21-1.65)and 1.33; 95% CI(0.61-2.52)), while the SMR for localized cancer survivors was similar to that in the general population (SMR=1.05; 95% CI(0.92-1.19)). The suicide rate of survivors who did not undergo surgery (mortality rate, 17.77 per 100,000 person-years; SMR=2.77; 95% CI(1.86-3.98)) was much higher than those of survivors after surgery. Among those who received surgical treatment, survivors undergoing mastectomy (mortality rate, 9.42 per 100,000 person-years; SMR=1.34, 95% CI(1.16-1.55)) were more likely to commit suicide than survivors who received BCS (mortality rate: 6.62 per 100000 person; SMR=0.98, 95% CI(0.85-1.13)). As for chemotherapy and radiotherapy, there was no apparent difference between survivors who received chemotherapy or not, while a much higher suicide rate was observed in survivors who did not receive radiotherapy than that in those who have received radiotherapy (mortality rate: 10.54 vs 6.09 per 100,000 person-years; SMR: 1.57(1.38-1.78)vs 0.88(0.75-1.02)) (**Table 1**).

### Suicide incidence in breast cancer subtypes

Suicide rates varied in survivors with different histologic subtypes. While survivors with infiltrating duct and lobular cancer got the highest SMR of 1.30(0.90-1.82), the lowest SMR of 1.01 (0.68-1.45) was observed in invasive lobular cancer survivors. As molecular subtype data were available in survivors diagnosed after 2010, the difference of suicide incidence between different molecular subtypes were identified. A total of 153 suicide cases occurred in survivors diagnosed from 2010 to 2017, HER-2 subtype survivors had the highest risk of suicide (mortality rate, 16.76 per 100,000 people and SMR, 2.21; 95% CI(0.14-3.86)), followed by the TNBC subtype survivors (mortality rate, 14.33 per 100,000 people and SMR, 1.98; 95% CI(0.24-2.99)) (**Table 2**).

**Table 2:** Suicide rates, according to breast cancer sub-types, female breast cancer survivors.

Variable		No. of patients,n (%)	Person-years	No. of deaths,n (%)	Mortalityrate (per 100,000 person-years)	SMR (95%CI)
Histology subtype*	IDC	501,196 (78.50)	3,963,387.4	328 (79.23)	8.28	1.20 (1.07-1.34)
	ILC	56,832 (8.90)	431,924.99	29 (7.00)	6.71	1.01 (0.68-1.45)
	IDC+ILC	42,766 (6.70)	381,347.95	34 (8.21)	8.92	1.30 (0.90-1.82)
	Others	37,753 (5.91)	302,533.18	23 (5.55)	7.60	1.15 (0.73-1.73)
						
Molecular subtype*	Luminal A	218,576 (67.94)	1,124,427.61	96 (62.75)	8.54	1.15 (0.93-1.41)
	Luminal B	35,288 (10.97)	175,657.00	11 (7.19)	6.26	0.80 (0.40-1.44)
	HER-2	14,908 (4.63)	71,600.94	12 (7.84)	16.76	2.21 (0.14-3.86)
	TNBC	33,233 (10.33)	153,542.29	22 (14.38)	14.33	1.98 (0.24-2.99)
	Unknown	19,712 (6.13)	98,956.75	12 (7.84)	12.13	1.69 (0.87-2.95)

*SMR, standardized mortality ratio; CI, confidence intervals; IDC, infiltrating duct cancer; ILC, infiltrating lobular cancer; IDC+ILC, infiltrating duct and lobular cancer; TNBC, triple negative breast cancer.

*Histology subtype information used data between 2000 and 2017; Molecular subtype information used data between 2010 and 2017.

### Suicide incidence after diagnose

The relative increase in suicide incidence among female breast cancer survivors was the highest within the first year after initial diagnosis and decreased with longer follow-up time. Between five to ten years after diagnosis, the suicide rate of female breast cancer survivors decreased to the level similar to that of the general population (mortality rate, 6.30 per 100,000 person-years and SMR, 0.91; 95% CI (0.74-1.12)), while it increased again over 10 years since diagnosis (mortality rate, 7.70 per 100,000 person-years and SMR, 1.15; 95% CI (0.89-1.45)) (**Table 3**).

**Table 3:** Suicide rates, according to time since diagnosis, female breast cancer survivors, 2000 to 2017.

Time since diagnosis	No. of patients,n (%)	Person-years	No. of deaths,n (%)	Mortality rate (per 100,000 person-years)	SMR (95%CI)
<1year	638,547	623,141.26	60 (14.50)	9.63	1.43 (1.09-1.84)
1-5 year	610,475	2,065,641.56	191 (46.14)	9.25	1.35 (1.16-1.55)
5-10year	408,786	1,507,099.18	95 (22.95)	6.30	0.91 (0.74-1.12)
≧10year	209,921	883,311.51	68 (16.43)	7.70	1.15 (0.89-1.45)
Total	638,547	5,079,193.52	414	8.15	1.19 (1.08-1.31)

SMR, standardized mortality ratio; CI, confidence intervals.

### Risk factors for suicide in breast cancer patients

**Figure 2:**
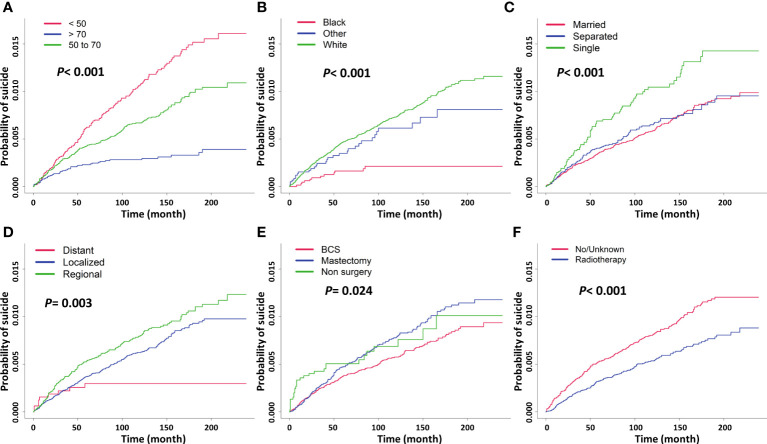
Cumulative incidence estimates of suicide among female breast cancer survivors by different risk factors, **(A)** age group, **(B)** race/ethnicity, **(C)** marital status, **(D)** stage, **(E)** surgery type, **(F)** radiotherapy.

Age, race, marital status, stage, surgery type, and radiotherapy were analyzed to be significantly associated with cumulative incidence of suicide by the Gray’s test. The CIF curves were plotted according to these risk factors (**Figure 2**). The result of the CIF subgroup analysis illustrated that a higher risk of suicide occurred in survivors aged <50 years, being white race, unmartial status (single and separated), regional stage, without surgery, and no radiotherapy performed. The Gray’s test was also performed on molecular subtypes using data on survivors diagnosed from 2010 to 2017 and a CIF curve was plotted (**Figure S1**). As for the survivors who were diagnosed with HER-2 and TNBC breast cancer, their cumulative incidence of 5-year suicide was 0.0008 and 0.0007, respectively.

Further multivariable Fine-Gray competing risk model revealed older age (50-70 vs <50: HR=0.65, 95% CI:0.52-0.80; >70 vs <50: HR=0.22, 95% CI:0.15-0.32), non-white race (black vs white: HR= 0.20, 95% CI: 0.11-0.36; other race vs white: HR= 0.67, 95% CI: 0.46-0.97), and radiotherapy (Yes vs No/Unknown: HR= 0.62, 95% CI: 0.49-0.77) might be protective factors for suicide. Separated or single marital status (separated vs married: HR= 1.50, 95% CI: 1.16-1.94; single vs married: HR= 1.70, 95% CI: 1.31-2.20) and regional stage (distant vs regional: HR= 0.30, 95% CI: 0.14-0.63) were risk factors for suicide among female breast cancer survivors (**Table 4**).

**Table 4:** Adjusted hazard risk of suicide among female breast cancer survivors assessed by the Fine and Gray model.

Variable		2000-2017		2010-2017	
		HR (95%CI)	*P*-value	HR (95%CI)	*P*-value
Age group					
	0-49	Ref.		Ref.	
	50-69	0.65 (0.52-0.8)	< 0.001	0.61 (0.43-0.86)	5.30 E^-03^
	70+	0.22 (0.15-0.32)	< 0.001	0.13 (0.06-0.27)	< 0.001
Race/ethnicity					
	White	Ref.		Ref.	
	Black	0.20 (0.11-0.36)	< 0.001	0.21 (0.094-0.49)	< 0.001
	Other	0.67 (0.46-0.97)	0.032	0.56 (0.30-1.04)	0.066
Year of diagnosis					
	2000-2004	Ref.			
	2005-2009	1.13 (0.89-1.44)	0.33		
	2010-2014	1.12 (0.86-1.46)	0.39	Ref.	
	2015-2017	1.22 (0.86-1.74)	0.27	1.08 (0.76-1.55)	0.66
Residence					
	Metropolitan	Ref.		Ref.	
	Non-metropolitan	0.87 (0.63-1.21)	0.41	1.15 (0.69-1.93)	0.58
	Unknown	3.53 (0.85-14.54)	0.081		
Marital status					
	Married	Ref.		Ref.	
	Single	1.7 (1.31-2.21)	< 0.001	2.34 (1.58-3.46)	< 0.001
	separated	1.5 (1.16-1.94)	2.10 E^-03^	1.37 (0.86-2.19)	0.18
	Unknown	1.26 (0.8-1.99)	0.32	1.44 (0.78-2.69)	0.25
Laterality					
	Left-primary	Ref.		Ref.	
	Right-primary	1.02 (0.84-1.24)	0.82	0.79 (0.57-1.09)	0.15
	Unknown	1.46 (0.2-10.84)	0.71	2.67 (0.31-22.96)	0.37
Grade*					
	I	Ref.		Ref.	
	II	0.96 (0.73-1.28)	0.8	0.89 (0.56-1.43)	0.64
	III	1.07 (0.79-1.45)	0.67	1.25 (0.74-2.13)	0.41
	IV	1.12 (0.45-2.83)	0.81	1.96 (0.26-14.75)	0.51
	Unknown	0.85 (0.52-1.41)	0.54	0.76 (0.29-1.97)	0.57
Stage					
	Regional	Ref.		Ref.	
	Localized	0.81 (0.65-1.01)	0.061	0.68 (0.46-0.99)	0.047
	Distant	0.30 (0.14-0.63)	1.40E^-03^	0.32 (0.11-0.94)	0.038
	Unknown	1.15 (0.52-2.53)	0.061	1.80 (0.62-5.12)	0.28
Surgery*					
	None	Ref.		Ref.	
	BCS	0.76 (0.47-1.23)	0.26	0.68 (0.32-1.42)	0.31
	Mastectomy	0.74 (0.47-1.16)	0.19	0.62 (0.31-1.22)	0.17
	Unknown	1.98 (0.67-5.85)	0.22	1.95 (0.45-8.47)	0.37
Chemotherapy					
	No/Unknown	Ref.		Ref.	
	Yes	0.88 (0.7-1.09)	0.24	0.87 (0.59-1.28)	0.49
Radiotherapy					
	No/Unknown	Ref.		Ref.	
	Yes	0.62 (0.49-0.77)	< 0.001	0.56 (0.39-0.81)	1.90 E^-03^
Histologic subtype*					
	IDC	Ref.		Ref.	
	ILC	0.88 (0.59-1.29)	0.5	1.23 (0.7-2.18)	0.47
	IDC+ILC	1.16 (0.82-1.64)	0.41	1.16 (0.59-2.3)	0.67
	Other	0.94 (0.61-1.43)	0.76	1.05 (0.51-2.16)	0.89
Molecular subtype*					
	Luminal B			Ref.	
	Luminal A			1.7 (0.91-3.19)	0.095
	HER-2			2.53 (1.1-5.82)	0.029
	TNBC			2.11 (1.01-4.41)	0.048
	Unknown			1.8 (0.78-4.14)	0.17

* HR, hazard ratio; CI, confidence intervals; Grade I, Well differentiated; Grade II, Moderately differentiated; Grade III, Poorly differentiated; Grade IV, Undifferentiated; BCS, breast-conserving surgery; IDC, infiltrating duct cancer; ILC, infiltrating lobular cancer; IDC+ILC, infiltrating duct and lobular cancer; TNBC, triple negative breast cancer.

The multivariable Fine-Gray competing risk model was also performed on patients diagnosed after 2010 and molecular subtypes were included in the model. The result revealed that older age (50-70 vs <50: HR=0.61, 95% CI: 0.43-0.86; >70 vs <50: HR=0.13, 95% CI: 0.06-0.27), non-white race (black vs white: HR= 0.21, 95% CI: 0.094-0.49; other race vs white: HR= 0.56, 95% CI: 0.30-1.04), and radiotherapy (Yes vs No/Unknown: HR= 0.56, 95% CI: 0.39-0.81) were protective factors for suicide, while single marital status (single vs married: HR= 2.34, 95% CI: 1.58-3.46), regional stage (distant vs regional: HR= 0.32, 95% CI: 0.11-0.94; localized vs regional: HR= 0.68, 95% CI: 0.46-0.99), and HER-2 or TNBC breast cancer (HER-2 vs Luminal B (HR= 2.53, 95% CI: 1.10-5.82), TNBC vs Luminal B (HR= 2.11, 95% CI: 1.01-4.42)) were risk factors for suicide among female breast cancer survivors (**Table 4**).

### Nomogram to evaluate suicide probability

**Figure 3:**
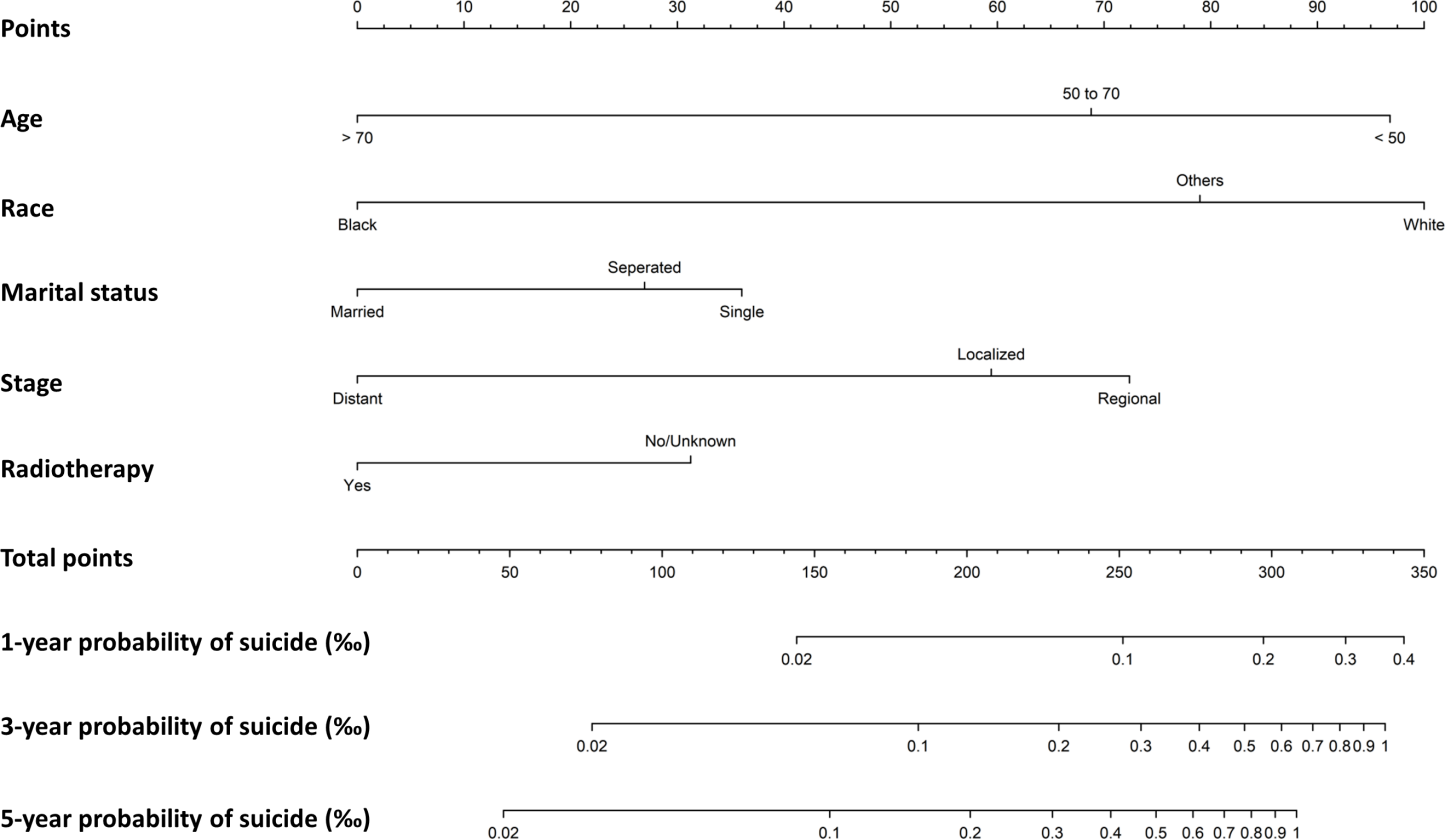
Nomogram to predict first-, three- and five-year probabilities of suicide for female breast cancer survivors.

A nomogram was generated on the basis of result from the multivariate Fine-Gray model using data on survivors diagnosed from 2000 to 2017 (**Figure 3**). The nomogram was used to show the corresponding score on the points row above the graph for each variable included in the model. All the single score of the variables were added to calculate the total score, and then straight lines were drawn to the bottom of the graph to estimate the probability of suicide at different time. The C-index of the nomogram prediction model was 0.62 (95%CI: 0.59-0.66). The calibration plot showed a high consistency between the predicted and the observed suicide events evaluating 1-year, 3-year, and 5-year probability of suicide (**Figure S2**).

## Discussion

In this population-based study, we presented a comprehensive analysis of suicide incidence and risk factors for female breast cancer survivors. The result indicated that the suicide rate of female breast cancer survivors was 8.15 per 100,000 person-years, and total SMR was 1.19 (95% CI: 1.08-1.31). Over time, the suicide rate among female breast cancer survivors increased, which could just be a reflection of the US population’s overall tendency (5). And female breast cancer survivors with younger age (less than 50 years old), white race, unmarried status, regional stage, no radiotherapy performed, and HER-2 or TNBC subtype were more likely to commit suicide according to the result of multivariable Fine-Gray competing risk model.

Several studies found sex differences in suicide rate in cancer survivors, in which suicide rate for females was much lower. Zaorsky et al. made a pan-cancer analysis of suicide and found a female to male mortality rate ratio of 9.22/50.28 per 100,000 person-years in cancer survivors (10). As for suicide rate in site-specific cancers, Yu et al. reported a mortality rate of 8.63 per 100,000 person-years in female leukemia patients, Guo et al. reported a mortality rate of 7.84 per 100,000 person-years in female kidney cancer patients, Chen et al. reported a mortality rate of 7.05 per 100,000 person-years in female liver cancer patients; the suicide mortality rates were all lower compared to male (17–19). What’s more, the incidence cases of male breast cancer is small and less than 1% of all breast cancers occurred in males, so we here only focused on female breast cancer (20). Compared to a previous study on suicide in breast cancer of both sexes, we here analyzed more clinical factors especially molecular subtypes (15). In addition, the method used in this study has considered survival time, making it possible to evaluate suicide at a specific time after diagnosis. Moreover, although both used data from the SEER database, data selection and study period were also different from the previous study. Thus, the findings of the two studies differed in some areas. For instance, the results of the previous study demonstrated that the nonwhite-nonblack race and earlier stage were risk factors, whereas the findings of our study showed that the white race was a risk factor in comparison to other races and the regional stage had a higher risk of suicide than the localized stage. The disparity can have been impacted by all the aforementioned reasons.

Most of the female breast cancer survivors were between 50 to 70 years old, while suicide rate gradually decreased with age (20). The higher suicide rate was observed in younger survivors, which was consistent with previous study of suicide in breast cancer survivors (15). And younger age was also identified as an independent risk factor for suicide through multivariate Fine-Gray model. The higher suicide rate and risk for younger survivors might be related to their outlook about life and death, and psychological condition such as the presence of depression (21, 22). As reported, younger women with breast cancer may fare worse in both physical and psychosocial dimensions after breast cancer diagnosis, when compared to older women who felt more familiarized with disease and death (21). Race differences in suicide also existed in female breast cancer survivors. Although SMR for white race patients was lower which may be influenced by the total population number, the mortality rate of white race patients was higher and white race was recognized as a risk factor compared to black race. These results might be related to differences in genes, religious beliefs, and economic and social conditions (23–25). Higher suicide rate and risk were observed in survivors who never married and survivors who were alone compared to married survivors. And unmarried status was an independent risk factor for suicide in individuals with breast cancer (separated vs married: HR= 1.50, CI: 1.16-1.94; single vs married: HR= 1.70, CI: 1.31-2.20). These results were consistent with findings in other cancer types and may be explained by the situation of lower socioeconomic status, less emotional support and less social attention of the unmarried survivors (18, 26–29).

With regard to specific clinical features of breast cancer, several findings should be noteworthy. As reported by several studies that histologic subtype was not significantly associated with suicide risks among non-small cell lung cancer patients and colorectal cancer patients, we here got a similar finding from multivariate analysis although a lower mortality rate was observed in ILC patients (30, 31). However, significant differences existed in molecular subtypes. Breast cancer could be divided into four molecular subtypes which provide insights into new treatment strategies and influenced the recovery management of patients (32). Based on our study, suicide mortality rate and risk were also different for patients stratified by molecular subtypes. Among them, suicide rates of HER2 and TNBC survivors were significantly higher than Luminal A and B breast cancer survivors; the suicide risk for HER2 and TNBC subtypes was significantly higher than Luminal A and B. The higher suicide rate and risk for these two subtypes may be due to the malignancy degree and insensitive to adjuvant therapy. HER-2 and TNBC subtypes were more likely to relapse and metastasis, which would bring physical disability and economic burden to survivors (33–35). Distant stage was demonstrated as a protective factor compared with regional stage. We hypothesized that this may be explained by the worse prognosis and higher mortality in survivors with distant stage, who anticipated dying from their disease rather than from alternative reasons of death like suicide. Radiotherapy was performed to ensure that all of the cancerous cells were destroyed, minimizing the possibility of breast cancer recurrence especially for metastatic or unresectable breast cancer patients (36). The result here demonstrated that radiotherapy was a protective factor against suicide, which may emphasize the use of radiotherapy in improving survival and life quality.

Our study provided a nomogram evaluating the probability of suicide deaths for female breast cancer survivors. The nomogram model is a promising tool that could be easily used and interpreted by clinicians to assess suicide risks in female breast cancer patients. And for individual patients with high risk factors, the suicide probability at specific time was shown in the nomogram. In comparison with other cancer studies involving nomogram models of suicide, our study here generated the model from competing risk model, while other models were built on the basis of Cox regression model which ignored the influence of other cause of death (37, 38).

This study has some limitations. First, information about other important risk factors for suicide, such as complications of cancer and treatment, quality of life, social support, were not available. Second, incomplete information on psychological status, depression and previous suicide attempts, made it impossible to assess the association between these factors with suicide. Recurrence information was not accessible, which was a crucial clinical feature and may affect the final suicide choice. Third, data used in this study was limited to the US population. Further, with the use of public available data in this study, the prediction nomogram model should be validated in outer dataset to test the effectiveness.

## Conclusion

In conclusion, younger age, white race, unmarried status, regional stage, no radiotherapy performed, and HER-2 or TNBC subtypes were independent indicator of suicide in female breast cancer survivors. The nomogram prediction model would help to evaluate suicide probability for high-risk survivors. More measures should be taken on survivors with high risk factors by the clinicians and family members to prevent suicide.

## Data availability statement

The original contributions presented in the study are included in the article/**Supplementary Material**. Further inquiries can be directed to the corresponding author.

## Author contributions

WR conceived and designed the study. JS and YY collected the data, developed the analytic pipeline, and wrote the manuscript. YG made literature research and prepared manuscript. All authors contributed to and approved the final version of the manuscript.

## Acknowledgments

The authors would like to thank SEER for open access to the database.

## Conflict of interest

The authors declare that the research was conducted in the absence of any commercial or financial relationships that could be construed as a potential conflict of interest.

## Publisher’s note

All claims expressed in this article are solely those of the authors and do not necessarily represent those of their affiliated organizations, or those of the publisher, the editors and the reviewers. Any product that may be evaluated in this article, or claim that may be made by its manufacturer, is not guaranteed or endorsed by the publisher.

## Supplementary material

Supplementary Figure 1 Cumulative incidence estimates of suicide among female breast cancer survivors by molecular subtypes using data on survivors diagnosed from 2010 to 2017.

Supplementary Figure 2 Calibration plot showing the predictive performance of the nomogram.
