# Supplementary figures and images for: Suicide risk among female breast cancer survivors: A population–based study
Source: Front Oncol. 2022 Nov 24;12:986822. doi: 10.3389/fonc.2022.986822 (PMC9731673; doi:10.3389/fonc.2022.986822)

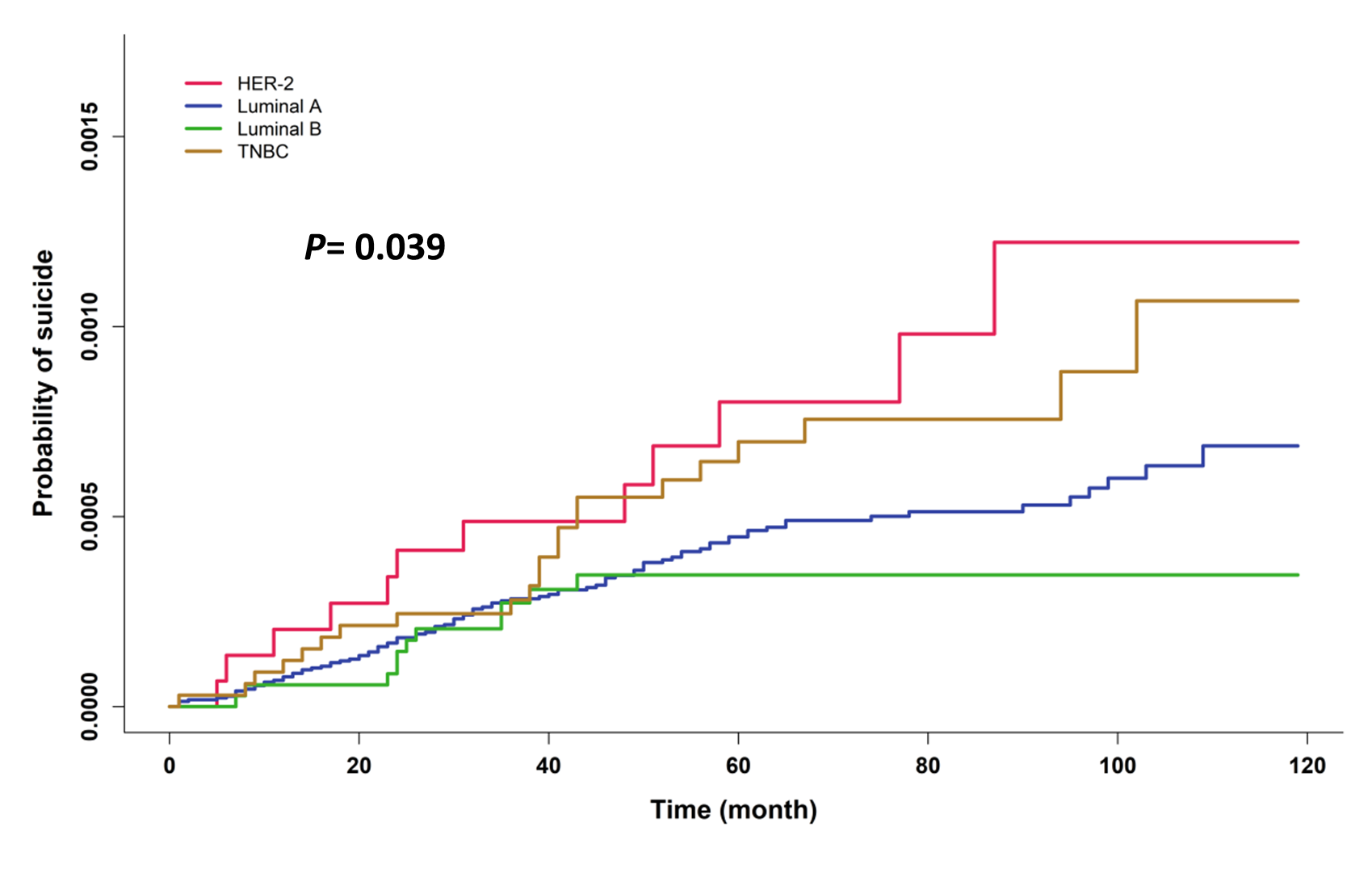

Supplement: Supplementary Figure 1 — Cumulative incidence estimates of suicide among female breast cancer survivors by molecular subtypes using data on survivors diagnosed from 2010 to 2017. [file Image_1.tif]

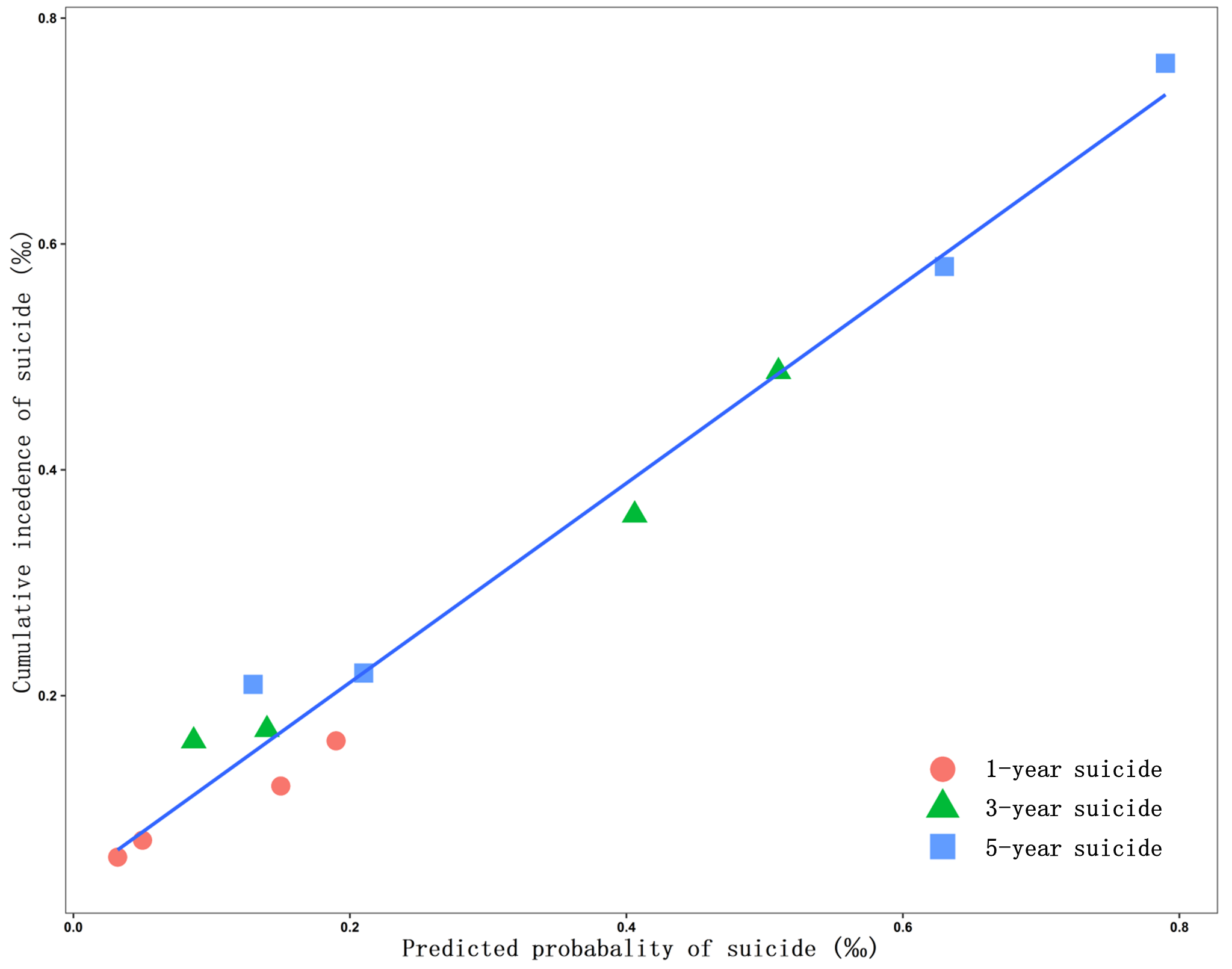

Supplement: Supplementary Figure 2 — Calibration plot showing the predictive performance of the nomogram. [file Image_2.tif]
