# Supplementary material for: Suicide risk among female breast cancer survivors: A population–based study
Source: Front Oncol. 2022 Nov 24;12:986822. doi: 10.3389/fonc.2022.986822 (PMC9731673; doi:10.3389/fonc.2022.986822)
Supplement: Supplementary file 3 [file Table_1.docx]

Table S1. Suicide rates of female breast cancer patients in different age groups.

| Age | No. of deaths, n (%) | Mortality rate | SMR (95%CI) |
| --- | --- | --- | --- |
| 0-29 | 3 (0.72) | 9.99 | 1.41(0.32-4.49) |
| 30-39 | 34 (8.21) | 11.57 | 1.54(0.97-1.96) |
| 40-49 | 125 (30.19) | 11.54 | 1.23(1.02-1.46) |
| 50-59 | 121 (29.22) | 8.62 | 1.10(0.91-1.31) |
| 60-69 | 84 (20.29) | 6.93 | 1.27(1.01-1.57) |
| 70-79 | 32 (7.73) | 4.27 | 1.02(0.70-1.44) |
| 80+ | 15 (3.62) | 4.88 | 1.31(0.73-2.15) |
